# Overcoming therapeutic inertia in LDL cholesterol-lowering treatment with education and simplified treatment algorithms

**DOI:** 10.1007/s12471-024-01863-x

**Published:** 2024-03-21

**Authors:** Maarten J. G. Leening

**Affiliations:** Departments of Cardiology, Radiology, and Epidemiology, Erasmus MC—University Medical Center Rotterdam, Rotterdam, The Netherlands

Cumulative exposure to atherogenic lipoproteins, including low-density lipoprotein cholesterol (LDL-C), plays a central role in the initiation and progression of atherosclerosis [1]. As such, maintaining lifelong optimal lipid levels is an important target for prevention of atherosclerotic cardiovascular disease (ASCVD). Healthcare professionals have 2 key parts to play: identifying individuals with (genetically) elevated LDL‑C and/or increased risk of ASCVD and, subsequently, optimising lipids through lifestyle interventions and pharmacological treatment. In this issue of the *Netherlands Heart Journal*, 2 Dutch studies highlight opportunities for improvement in ASCVD prevention related to identifying very high-risk individuals and optimising LDL-C-lowering treatment [2, 3].

## Knowledge gaps

Ibrahim and colleagues surveyed 221 Dutch general practitioners on their knowledge of familial hypercholesterolaemia [2]. This condition is characterised by lifelong genetically elevated LDL‑C levels and consequently very high ASCVD risk, often resulting in premature ASCVD if left untreated. Most Dutch general practitioners knew what defines familial hypercholesterolemia and which lipid profiles should trigger considering this diagnosis. However, it is noteworthy that only 14% of them correctly identified the associated ASCVD risks of lifelong very high LDL‑C levels, which is critical to an informed discussion on initiation and intensification of LDL-C-lowering treatment.

## Therapeutic inertia and structured LDL-C lowering

Omar Khader and colleagues evaluated a stepwise, structured LDL-C-lowering strategy in 999 Dutch patients with a recent acute coronary syndrome (ACS) and a history of ASCVD and/or diabetes in the PENELOPE study [3]. The primary endpoint of the study was achieving the Dutch guideline-directed LDL‑C goal of ≤ 1.8 mmol/L with generic oral lipid-lowering therapy (i.e. statins and/or ezetimibe). In total, 92% of the patients (915/999) completed the study, of whom 89% (836/915) met the primary endpoint.

Two important observations can be made from this study. First, despite a history of ASCVD (74%) and/or diabetes (46%) at the time of the index ACS, 28% of the patients were not using a statin, whereas only 3.5% reported a statin intolerance. This implies either poor persistence of statin use on the patient’s side or therapeutic inertia on the healthcare provider’s side before the index ACS. Therapeutic inertia is the failure to initiate or intensify treatment when treatment goals are not met, and is an established barrier to improving patient care and clinical outcomes [4].

Second, the observed proportion of patients at LDL‑C goal in the PENELOPE study (95% at the end of the study) is at odds with real-world multicentre registries. The EUROASPIRE V and DA VINCI registries demonstrated a disappointing LDL‑C goal attainment of 33% (in 2016) [5] and 62% (in 2018) [6] in the Netherlands, which is only marginally better than the reported European averages. This discrepancy suggests that following a structured intensive protocol to uptitrate lipid-lowering therapy, as employed in PENELOPE, likely has major effects on prescription patterns and goal attainment. Conversely, it also hints at substantial therapeutic inertia in contemporary clinical care.

## The road forward

The studies presented in this issue of the *Netherlands Heart Journal* provide key learning points for clinical practice and future research alike. First, registries to evaluate routine clinical practice remain relevant in 2024. Monitoring our real-world practice facilitates historical comparisons to identify trends and comparisons to other countries. We can thereby identify what aspects of care we must improve upon for our patients.

Next, continuous education of both general practitioners and cardiovascular specialists to expand their knowledge of dyslipidaemias, including familial hypercholesterolemia, treatment goals and effective treatment strategies, remains warranted. Lack of knowledge facilitates complacency and therapeutic inertia in everyday clinical practice, which is constrained by time and competing priorities [4].

Over the past decade, the focus of attention in lipid-lowering treatment has mostly revolved around novel therapies, such as PCSK9 inhibitors and bempedoic acid. However, as demonstrated by Omar Khader and colleagues, the vast majority of very high-risk patients with coronary heart disease can be adequately treated with affordable generic drugs. High-intensity statins and ezetimibe should remain the cornerstone of lipid-lowering treatment in every patient with ASCVD, with novel therapies reserved for select very high-risk patients. Challenges remain regarding optimal selection of these very high-risk patients: parenteral administration and costs of further LDL‑C lowering with non-generic therapies need to be weighed with other potent options to lower residual ASCVD risk, such as generic low-dose colchicine [7].

In parallel with the introduction of novel lipid-lowering therapies, international LDL‑C treatment goals have substantially changed for patients with ASCVD: the 2016 European Society of Cardiology (ESC) prevention guidelines recommended a treatment goal of ≤ 1.8 mmol/L (serving as the foundation of the prevailing Dutch CVRM guidelines), the 2019 ESC/EAS dyslipidaemia guidelines recommend a ≤ 1.4 mmol/L goal, and the 2023 ESC Guidelines for the management of ACS recommend a ≤ 1.0 mmol/L goal in select patients [8]. However, no single trial has been conducted in post-ACS patients to establish optimal LDL‑C goals. In fact, animal models, genetic studies, intracoronary imaging studies and an abundance of clinical data all point towards a linear or log-linear relation between attained LDL‑C levels and ASCVD outcomes, without safety concerns at extremely low attained LDL‑C levels. In short, the total body of evidence can be summarised with ‘the lower, the better’ and ‘the earlier, the better’. This message is reflected in the most recent, simplified ESC LDL‑C treatment algorithm by recommending immediate initiation of a high-dose, high-intensity statin and ezetimibe during hospital admission for ACS [8]. In line with the findings from the PENELOPE study, such a simplified practical treatment strategy will likely circumvent therapeutic inertia and result in better LDL‑C control than contemporary clinical practice.

In summary, the research conducted by Ibrahim and colleagues and Omar Khader and colleagues underscores the importance of continuous education and implementation of practical treatment recommendations to overcome therapeutic inertia in LDL‑C lowering in the Netherlands.
